# Data showing atherosclerosis-associated differentially methylated regions are often at enhancers

**DOI:** 10.1016/j.dib.2019.103812

**Published:** 2019-03-07

**Authors:** Michelle Lacey, Carl Baribault, Kenneth C. Ehrlich, Melanie Ehrlich

**Affiliations:** a Tulane Cancer Center, Tulane University Health Sciences Center, LA 70112, USA; b Department of Mathematics, Tulane University, New Orleans, LA 70118, USA; c Center for Bioinformatics and Genomics, Tulane University Health Sciences Center, USA; d Hayward Genetics Center, Tulane University Health Sciences Center, New Orleans, LA 70112, USA

**Keywords:** Atherosclerosis, DNA methylation, Smooth muscle, Monocytes, Enhancers, Differentially methylated regions (DMRs), DMR, differentially methylated region, athero hypermeth DMR, atherosclerosis-associated hypermethylated DMR, athero hypometh DMR, atherosclerosis-associated hypomethylated DMR, GO, gene ontology, PMD, percent methylation difference, ctl, control, mod, moderate, SkM, skeletal muscle, PBMC, peripheral blood mononuclear cells, enh, enhancer chromatin, prom, promoter chromatin, repr, repressed, txn chromatin, chromatin with the histone marks of actively transcribed chromatin

## Abstract

Atherosclerosis involves phenotypic modulation and transdifferentiation of vascular smooth muscle cells (SMCs). Data are given in tabular or figure format that illustrate genome-wide DNA methylation alterations in atherosclerotic vs. control aorta (athero DMRs). Data based upon publicly available chromatin state profiles are also shown for normal aorta, monocyte, and skeletal muscle tissue-specific DMRs and for aorta-specific chromatin features (enhancer chromatin, promoter chromatin, repressed chromatin, actively transcribed chromatin). Athero hypomethylated and hypermethylated DMRs as well as epigenetic and transcription profiles are described for the following genes: *ACTA2, MYH10, MYH11* (SMC-associated genes); *SMAD3* (a signaling gene for SMCs and other cell types); *CD79B* and *SH3BP2* (leukocyte-associated genes); and *TBX20* and genes in the *HOXA, HOXB, HOXC*, and *HOXD* clusters (T-box and homeobox developmental genes). The data reveal strong correlations between athero hypermethylated DMRs and regions of enhancer chromatin in aorta, which are discussed in the linked research article “Atherosclerosis-associated differentially methylated regions can reflect the disease phenotype and are often at enhancers” (M. Lacey et al., 2019).

**Table:** Specifications table

Subject area	*Biology*
More specific subject area	*Molecular biology, gene regulation, epigenetics*
Type of data	*Figures, tables, text file*
How data was acquired	*Downloaded publically available data from the UCSC genome browser*
Data format	*filtered, analyzed*
Experimental factors	*Bisulfite-seq profiles for atherosclerotic and control aorta were analyzed for differentially methylated regions (DMRs)*
Experimental features	*Original data obtained from UCSC genome browser and various hubs*
Data source location	*Data were collected and the DMRs determined at Tulane University, New Orleans, LA, USA, Longitude -90.071533, Latitude, 29.951065*
Data accessibility	*Data provided in this article and as Supplementary Tables*
Related research article	*The associated research article is available*[1]*.* M. Lacey et al., Atherosclerosis-associated differentially methylated regions can reflect the disease phenotype and are often at enhancers, Atherosclerosis, 280 (1) (2019) 183-191. https://doi.org/10.1016/j.atherosclerosis.2018.11.031.

**Table:** 

**Value of the data** • Genes with atherosclerosis-associated differentially methylated regions (DMRs) were defined from publicly available bisulfite-sequencing data by a stringent statistical analysis that revealed predominant hypermethylation but also atherosclerosis-relevant hypomethylation in atherosclerotic aorta and will be a source of disease-linked DMRs for further studies. • Data include figures and tables detailing the locations and percent methylation differences of hypermethylated and hypomethylated DMRs and whether these overlap enhancer or promoter chromatin in aorta, data that are valuable for understanding regulation of expression of genes involved in atherosclerosis. • The figures and tables are also a novel resource for understanding the tissue-specific epigenetics of many individual genes in normal aorta (e.g., smooth muscle cell-associated genes and HOX genes). • Data illustrate the often overlooked importance of enhancer regions to disease as well as to differentiation, which will aid future research on the regulation of gene expression.

## 1 Data

**Fig. 1:**
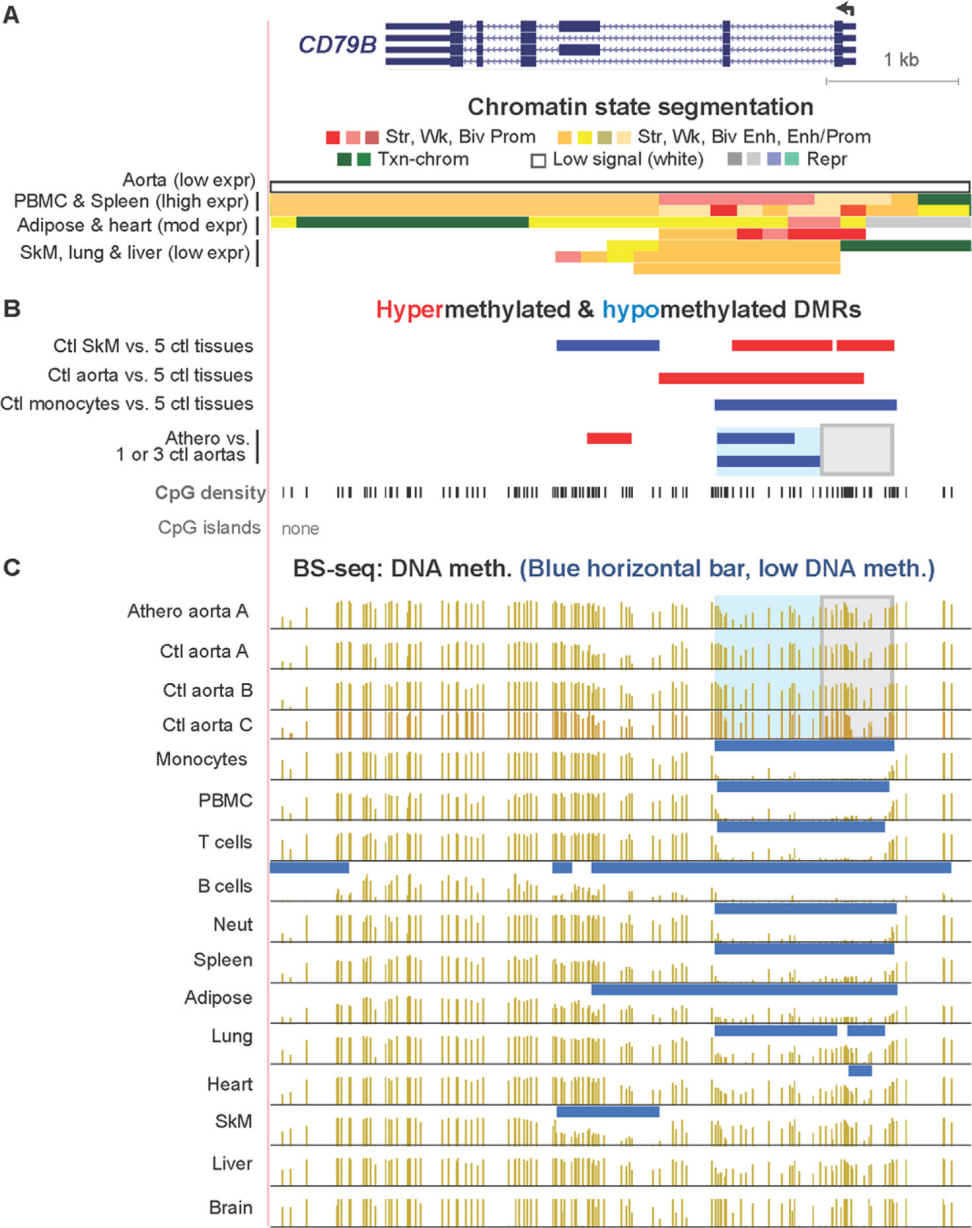
***CD79B*, a B-cell antigen-encoding gene, displays a leukocyte-associated hypomethylated region, which only partly overlaps an atherosclerosis-associated hypomethylated DMR.** (A) *CD79B* is aligned with chromatin state segmentation tracks labeled according to the indicated color code (RefSeq gene isoforms; chr17:62,005,196-62,010,606, hg19). Note the low signal for standard histone modifications throughout this region in aorta (white box). (B) Tissue-specific and atherosclerosis-related DMRs. The density of CpGs is shown; there were no CpG islands in this region according to the UCSC Genome Browser [5]. (C) Bisulfite-seq tracks are plotted as average % methylation at CpGs; blue horizontal bars, regions that display statistically significant hypomethylation relative to the rest of the same genome. Light blue highlighting, the region of atherosclerosis-associated hypomethylation overlapping monocyte- (and leukocyte-) hypomethylation; gray highlighting, the region that did not exhibit an athero hypometh DMR but did show monocyte- (and leukocyte-) hypomethylation. Ctl, control; SkM, skeletal muscle; brain, prefrontal cortex; heart, left ventricle; PBMC, peripheral mononuclear blood cells; neut, neutrophils; expr, expression; str, strong; wk, weak; biv, bivalent; prom, promoter; enh, enhancer; repr, repressed; Txn-chrom, chromatin with a type of histone modification seen in many actively transcribed chromatin regions (histone H3 lysine-36 trimethylation).

**Fig. 2:**
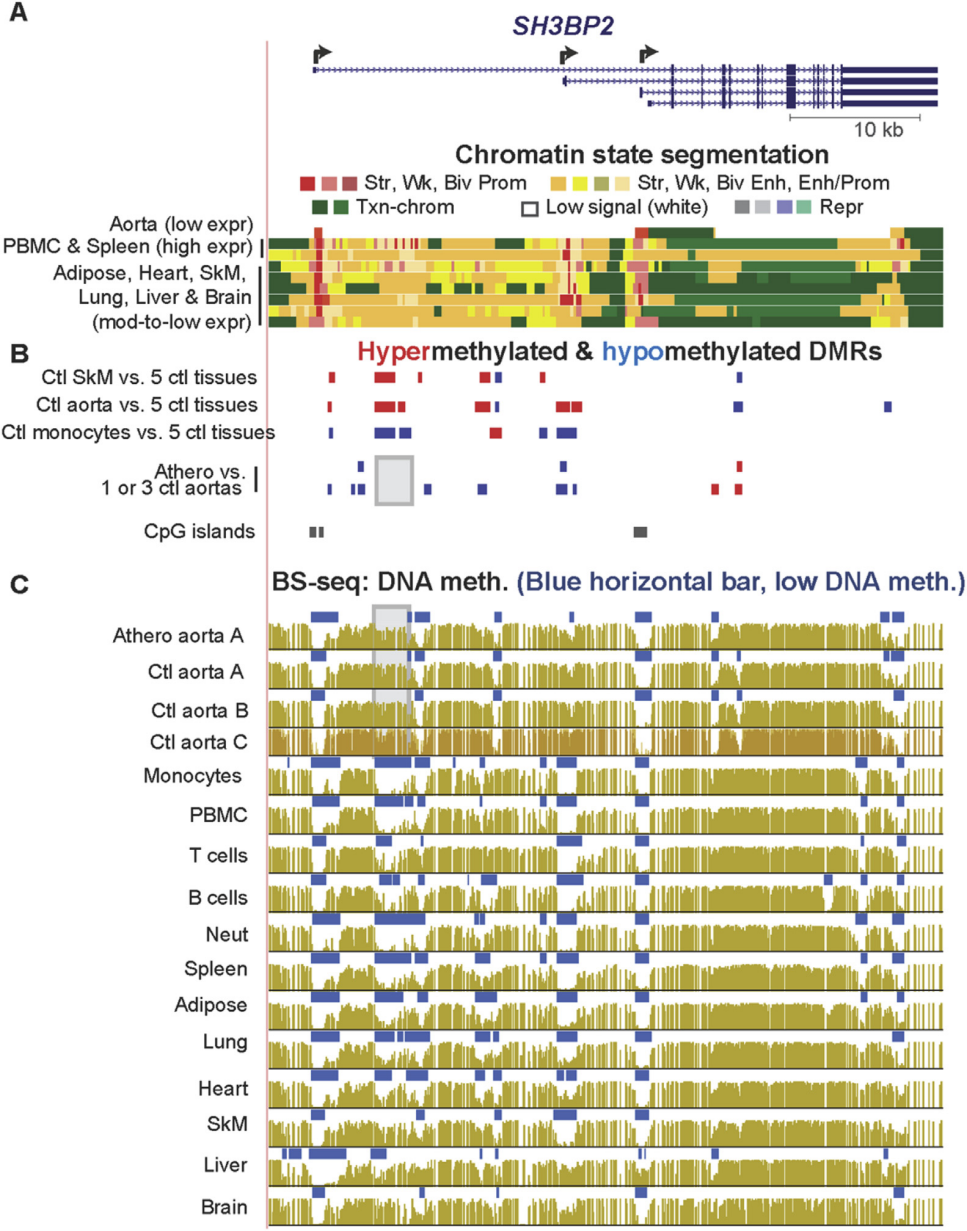
***SH3BP2*, which encodes a leukocyte-associated protein tyrosine kinase, lacks an atherosclerosis-associated hypomethylated DMR at one monocyte-associated hypomethylated DMR.** (A) Chromatin segmentation state of *SH3BP2*, which codes for a signaling protein involved in B cell development and signaling and in neutrophil functioning (chr4:2,791,263-2,843,297). (B) Tissue-specific and athero DMRs as in Fig. 1. (C) Bisulfite-seq profiles of DNA methylation with blue horizontal bars indicating statistically significant hypomethylation relative to the rest of the same genome except for Ctl aorta C, which did not have this kind of analysis in the public database. Gray highlighting, the region that did not exhibit an athero hypometh DMR but did show monocyte- (and leukocyte-) hypomethylation; mod, moderate.

**Fig. 3:**
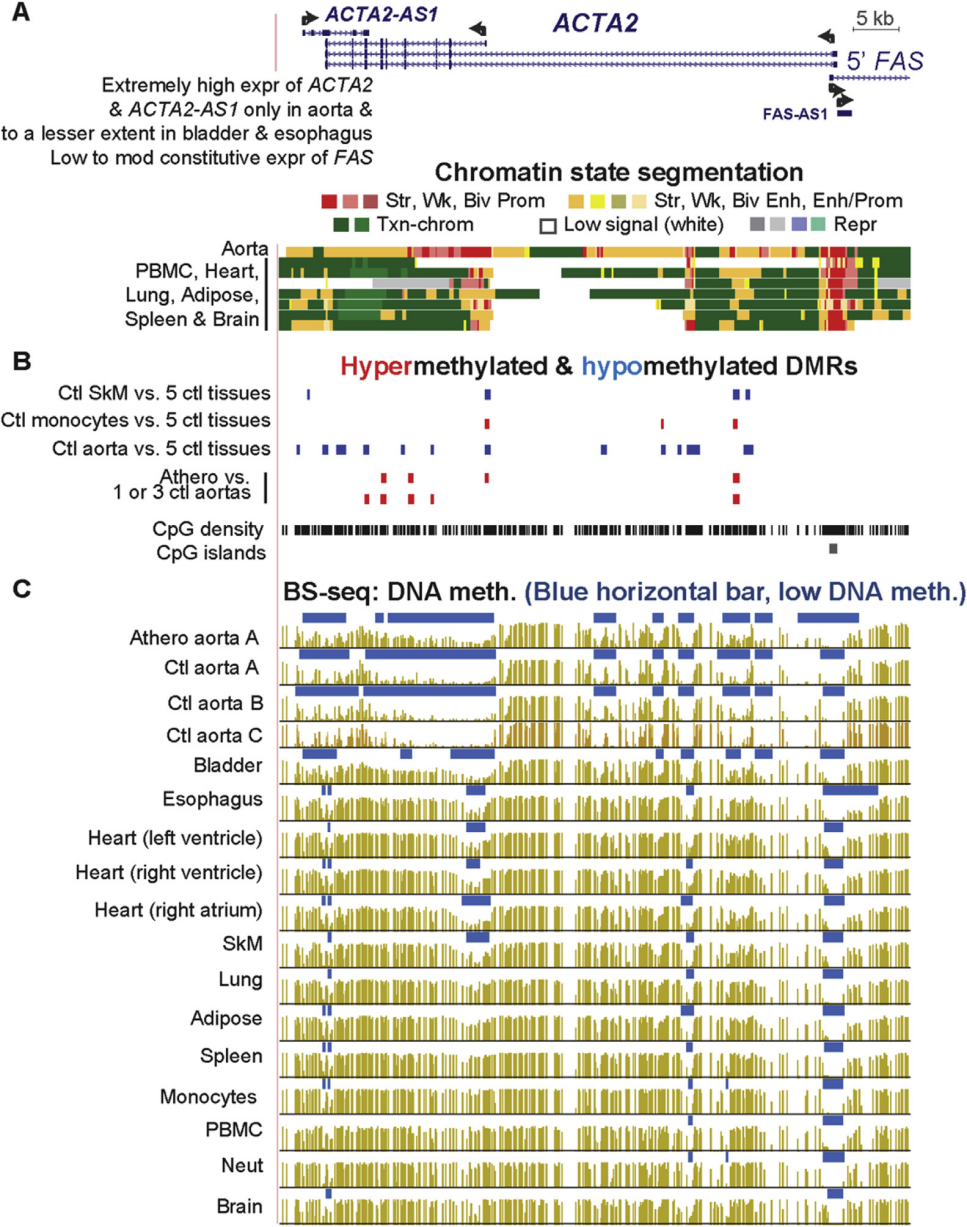
**Atherosclerosis-associated hypermethylation in the aorta α2 smooth muscle actin gene, *ACTA2,* encoding a marker for the SMC contractile phenotype.** (A) *ACTA2* RefSeq gene isoforms and neighboring genes; chromatin state segmentation (chr10:90,689,802-90,759,153). (B) Tissue-specific and atherosclerosis-associated DMRs. (C) Bisulfite-seq DNA methylation profiles.

**Fig. 4:**
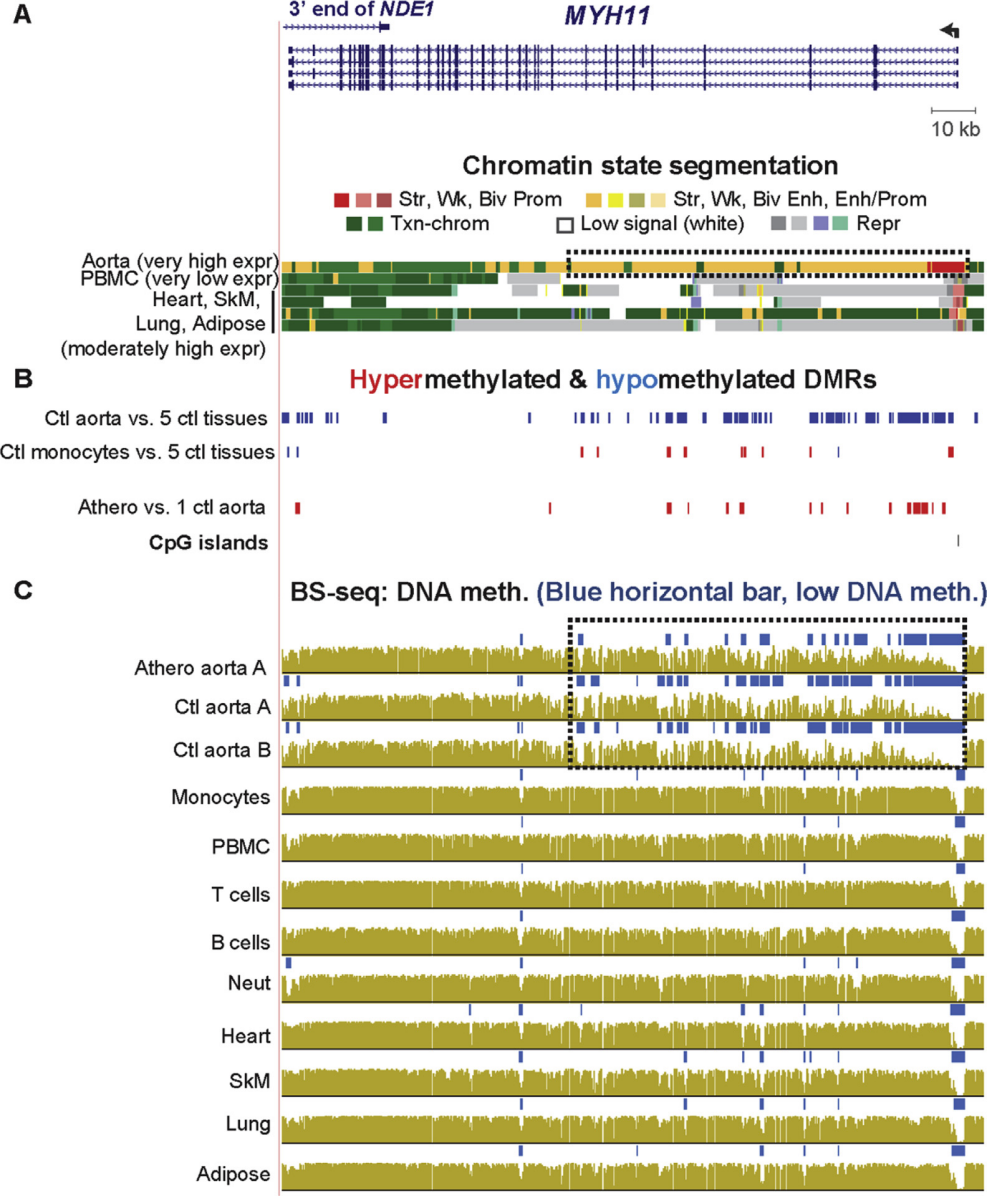
**Atherosclerosis-associated hypermethylation at an aorta super-enhancer in the smooth muscle myosin gene, *MYH11,*which encodes a marker for the SMC contractile phenotype.** (A) *MYH11* RefSeq gene isoforms and chromatin state segmentation (chr16:15,795,472-15,956,857). (B) Tissue-specific and atherosclerosis-associated DMRs. (C) Bisulfite-seq DNA methylation profiles. The *MYH11* gene neighborhood and a small minority of DNA regions had aberrantly low coverage and methylation in the bisulfite-seq track for Ctl aorta C (not shown) resulting in misleading DMR results for athero vs. 3 Ctl DMRs but not affecting the determination of DMRs from athero vs.1 Ctl DMR (the paired athero A/Ctl A aorta samples). The hypermethylation of the Athero aorta A sample is apparent in comparison to Ctl aorta B as well as Ctl aorta A. Dotted boxes, aorta super-enhancer region in aorta overlapping many athero hypermeth DMRs and aorta tissue-specific hypometh DMRs.

**Fig. 5:**
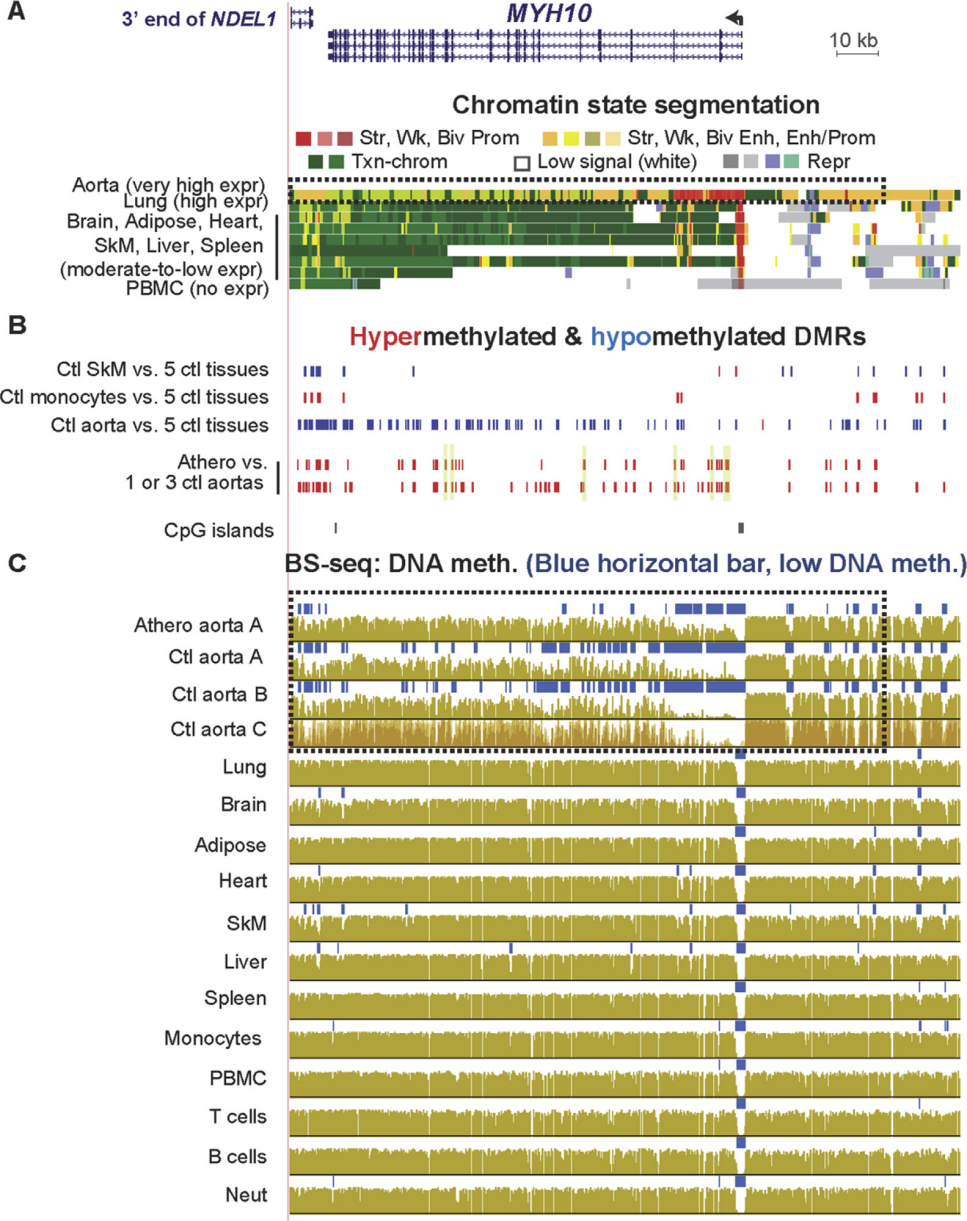
**Atherosclerosis-associated hypermethylation at an aorta super-enhancer in the *MYH10* myosin-encoding gene.** (A) *MYH10* RefSeq gene isoforms and chromatin state segmentation (chr17:8,362,804-8,617,136). (B) Tissue-specific and atherosclerosis-associated DMRs. (C) Bisulfite-seq DNA methylation profiles. Dotted boxes, super-enhancer region in aorta overlapping athero DMRs; yellow highlighting with black dot underneath in the DMR tracks, athero DMRs with PMD >40% (strongly hypermethylated). Note related *NDE1* and *NDEL1* genes downstream of MYH11 and MYH10, respectively.

**Fig. 6:**
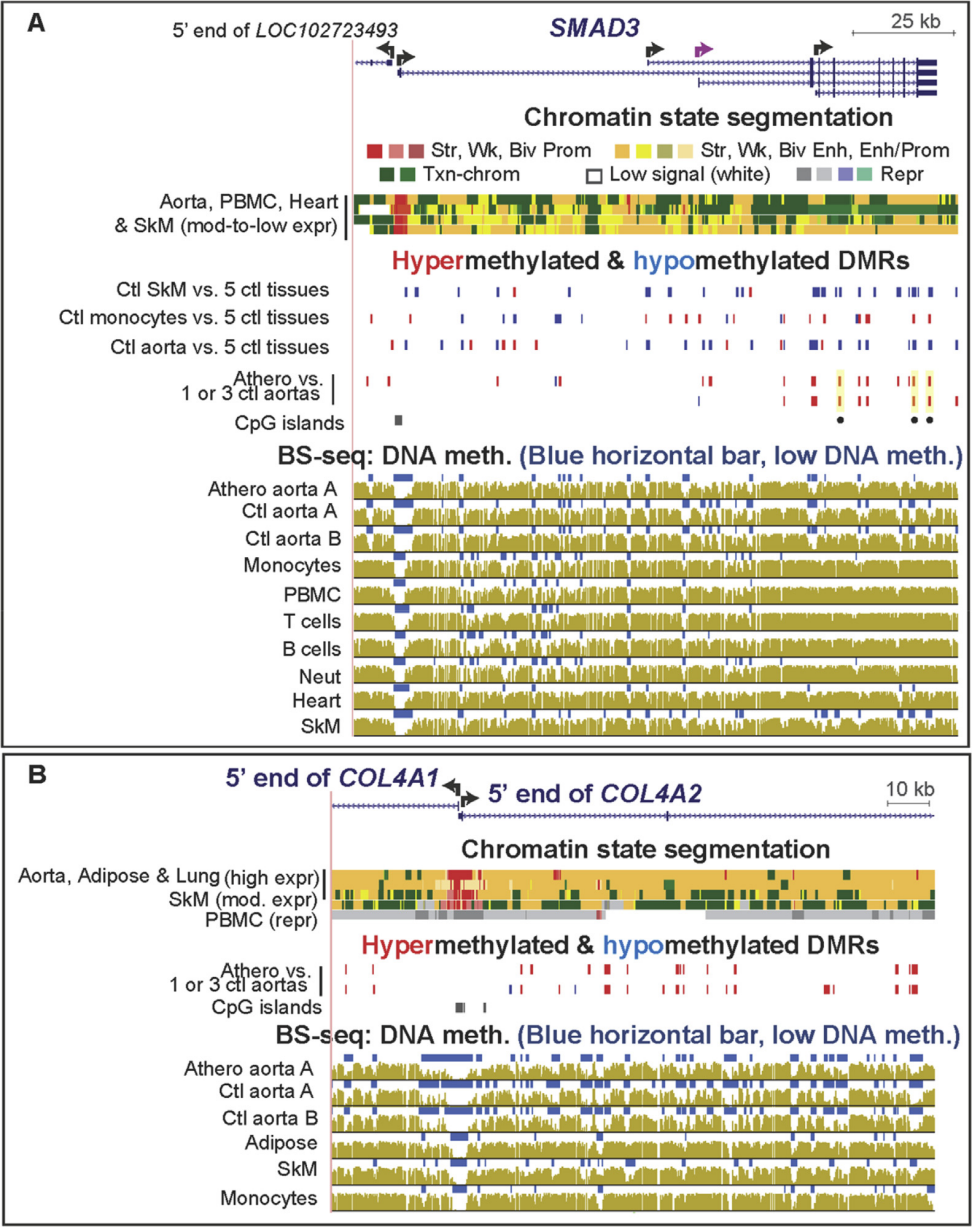
**Atherosclerosis-associated hypermethylation in the *SMAD3* signaling/transcription modulating gene and in the *COL4A2* collagen gene to which it is functionally linked.** (A) *SMAD3* (chr15:67,347,490-67,492,681), and (B) *COL4A2* (chr13:110,928,716-111,074,386) RefSeq gene isoforms, respectively with chromatin state segmentation, tissue-specific and atherosclerosis-associated DMRs and bisulfite-seq as in previous figures. Yellow highlighting with black dot underneath in the DMR tracks, athero DMRs with PMD >40% (strongly hypermethylated); broken purple arrow, alternative TSS that was used preferentially in aorta according to GTex Cufflinks analysis of RNA-seq profiles [7].

**Fig. 7:**
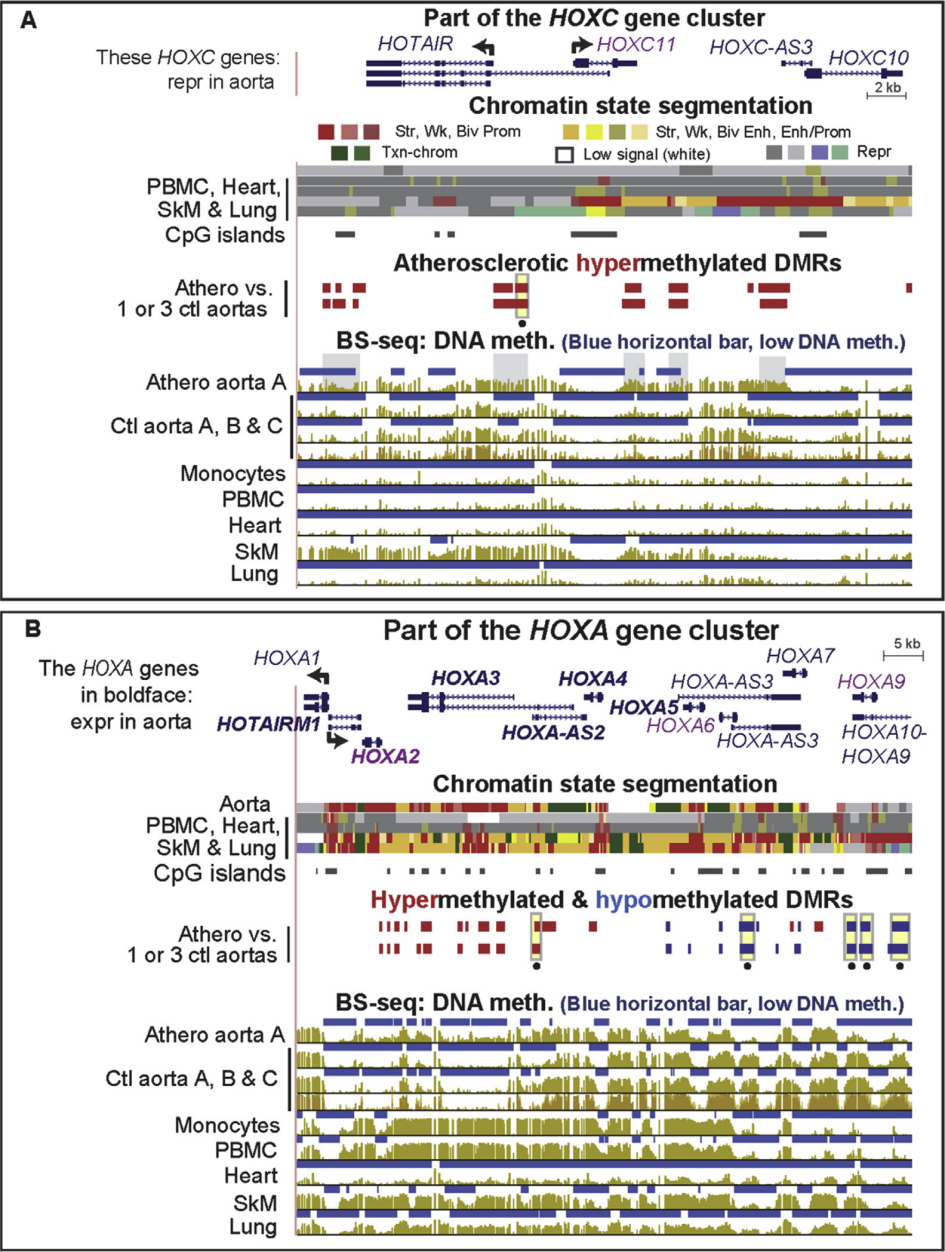
**High densities of atherosclerosis-associated DMRs span the *HOXC* and *HOXA* gene clusters.** (A) Part of the *HOXC* gene cluster (chr12:54,352,494-54,384,543). Gray highlighting in BS-seq athero aorta track, athero hypermeth DMRs that overlap regions of low methylation in monocytes. (B) Most of the *HOXA* gene cluster (chr7:27,131,681-27,209,593). Genes in purple font, these genes had their athero hypermeth or hypometh status previously determined at a few CpGs by Illumina microarray assays alone and with pyrosequencing assays on 15 matched pairs of atherosclerotic and control aorta with results consistent with those shown [2].

**Fig. 8:**
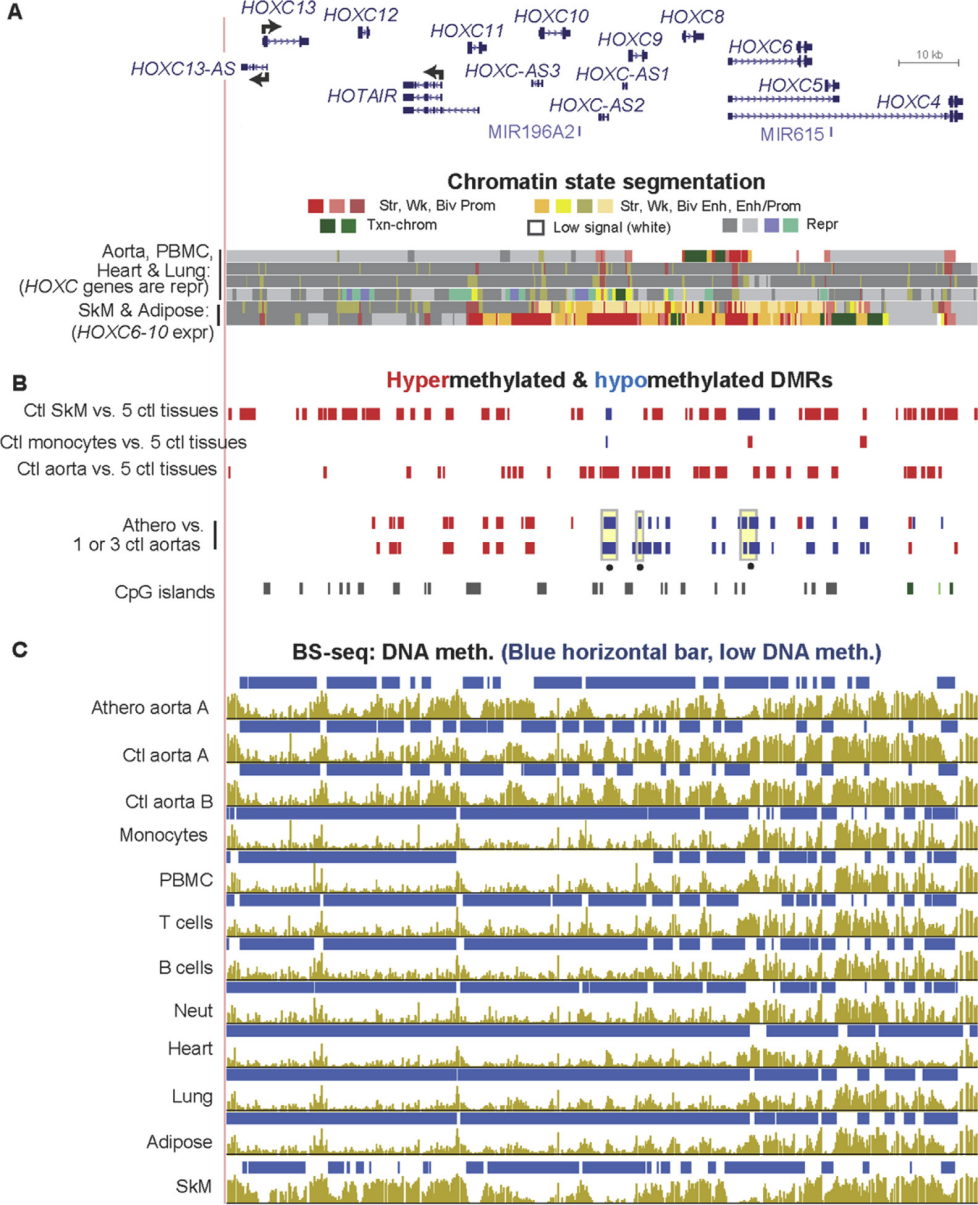
**The *HOXC* gene cluster displays predominantly atherosclerosis-associatedDNA hypermethylation in half of the cluster and athero hypomethylation in the other half.** (A) RefSeq gene isoforms and chromatin state segmentation (chr12:54,326,508-54,452,368). (B) Tissue-specific and atherosclerosis-associated DMRs. (C) Bisulfite-seq DNA methylation profiles. Yellow highlighting with black dot underneath in the DMR tracks, athero DMRs with PMD <40% (strongly hypomethylated). Note that there are regions of low leukocyte DNA methylation that overlap athero hypermeth DMRs and, therefore, monocyte infiltration could not explain these DMRs.

**Fig. 9:**
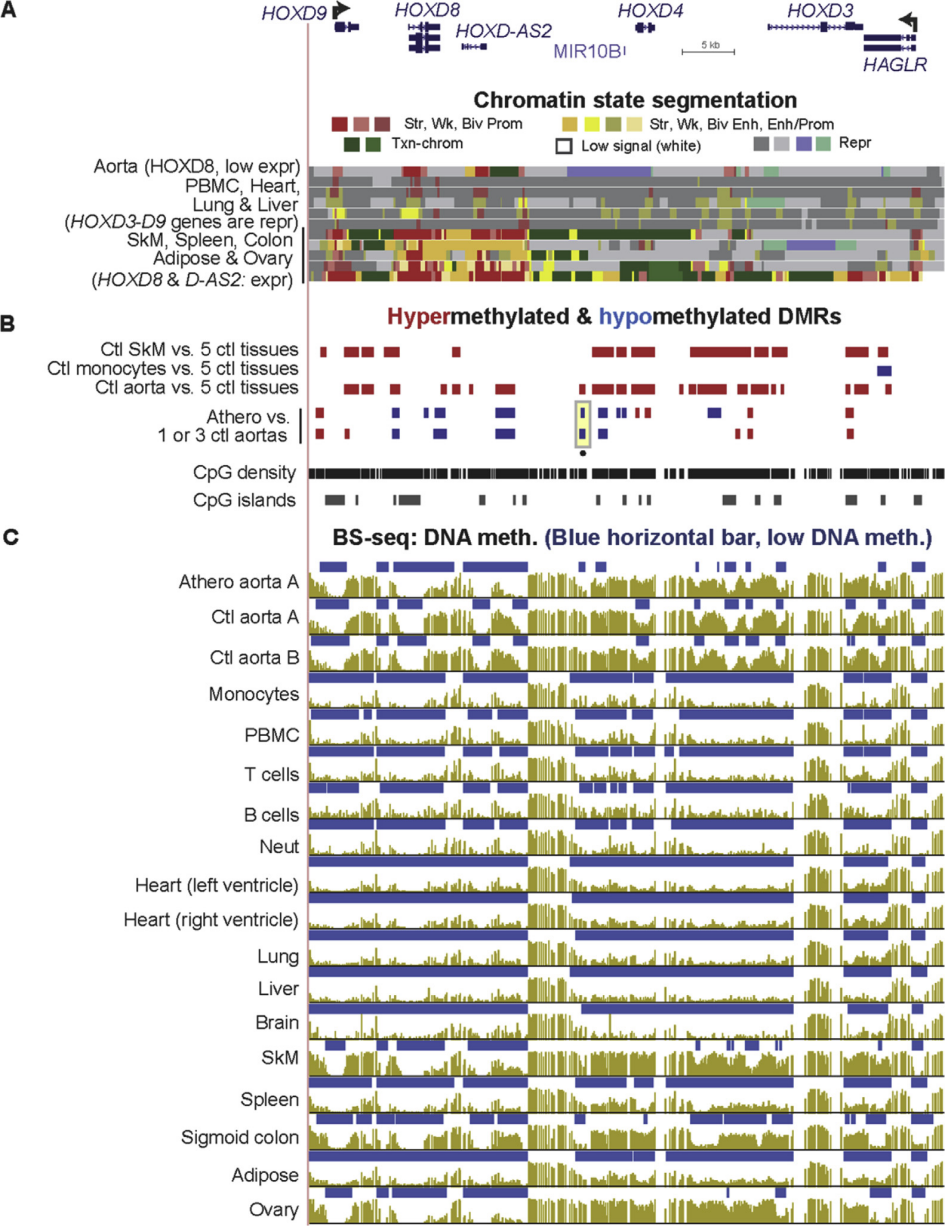
**The *HOXD* gene cluster displays many subregions exhibiting atherosclerosis-associated DNA hypermethylation or hypomethylation.** (A) RefSeq gene isoforms and chromatin state segmentation (chr2:176,984,957-177,045,568). (B) Tissue-specific and atherosclerosis-associated DMRs. (C) Bisulfite-seq DNA methylation profiles. Note that the athero hypermeth DMRs are in regions of low leukocyte DNA methylation and, thus, cannot be explained by monocyte infiltration.

**Fig. 10:**
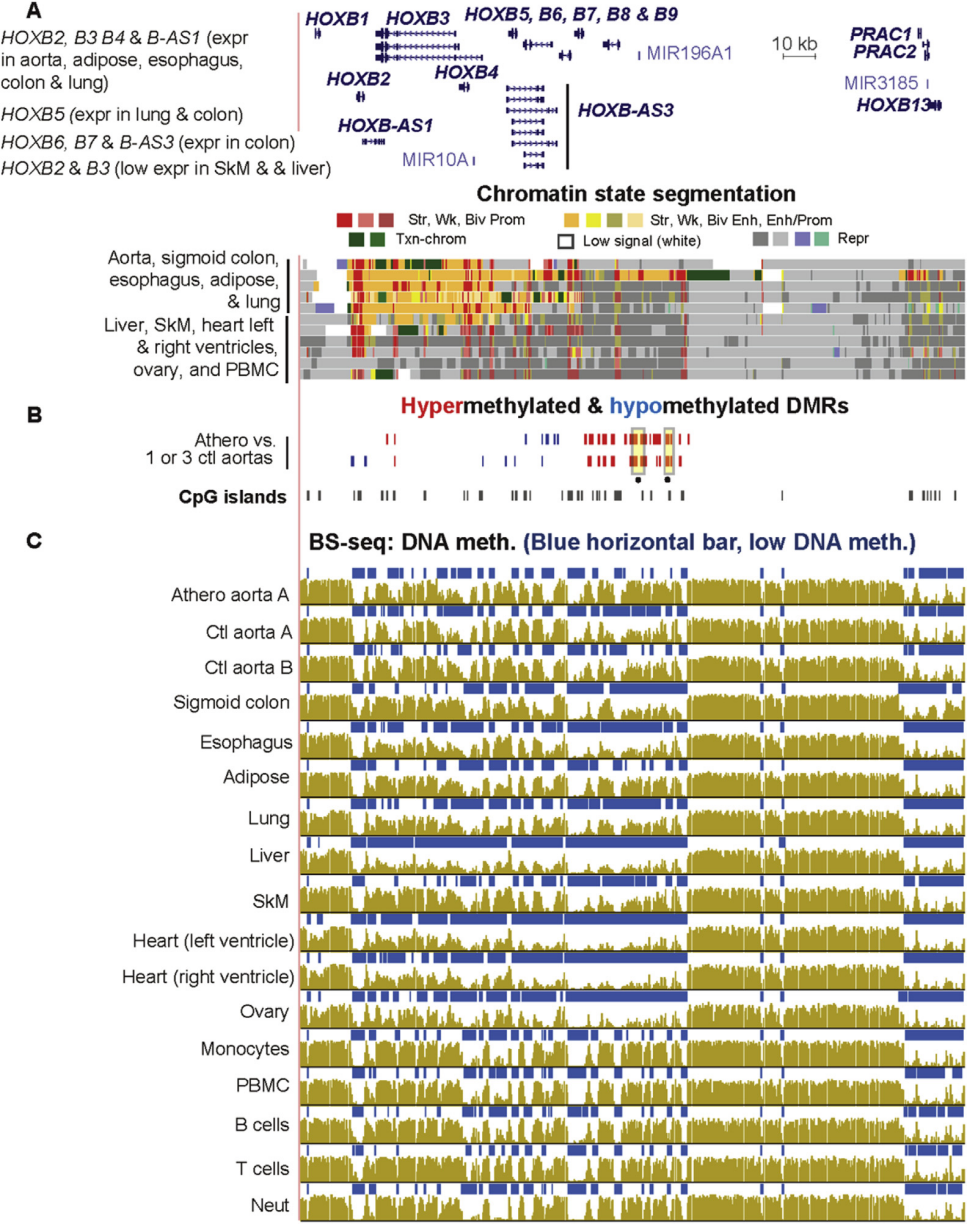
**The *HOXB* gene cluster displays a subregion exhibiting atherosclerosis-associated DNA hypermethylation and another with mostly athero hypomethylation.** RefSeq gene isoforms (chr17:46,602,205-46,813,770) with chromatin state segmentation, DMRs and bisulfite-seq as in previous figures.

**Fig. 11:**
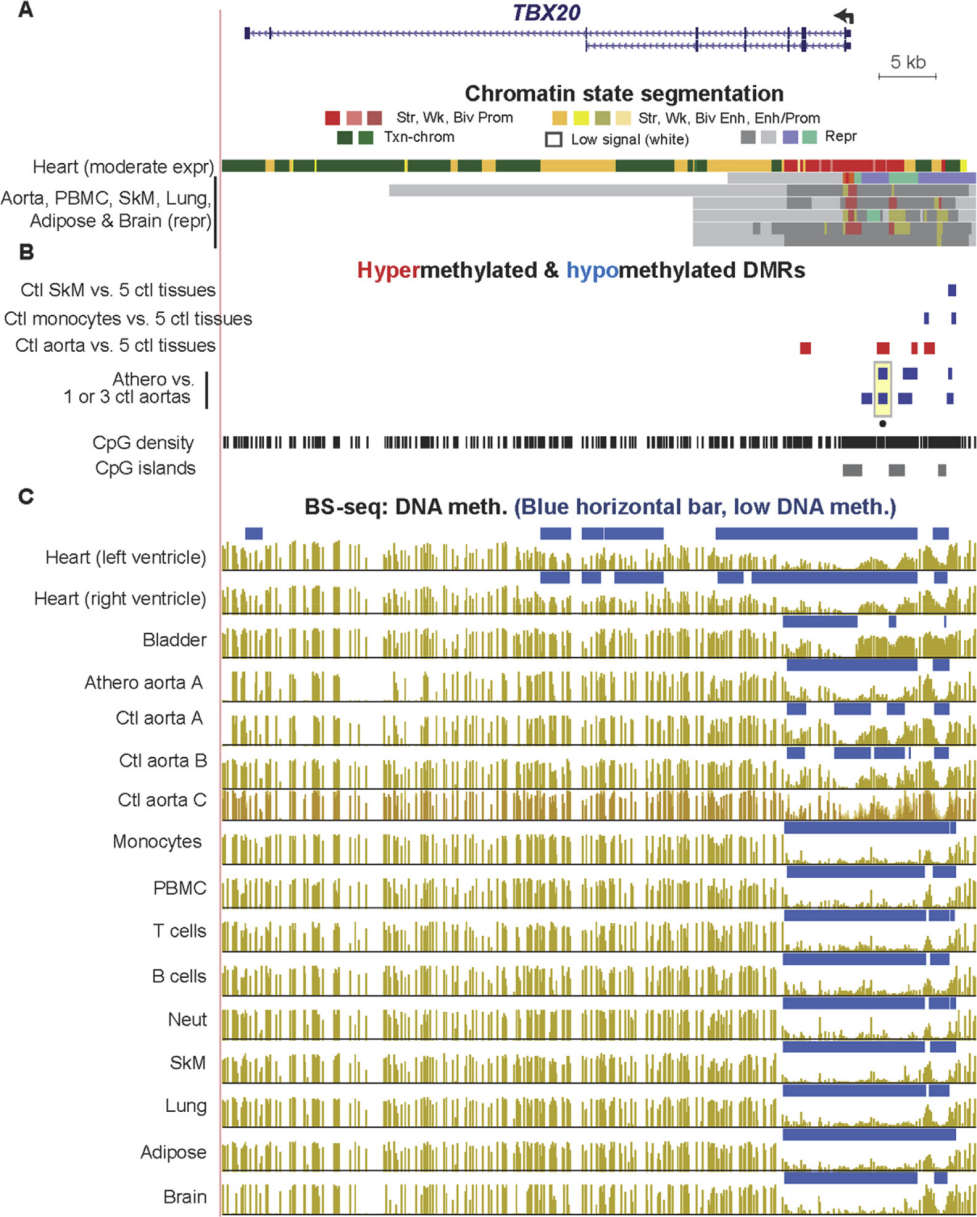
**Atherosclerosis-associated hypomethylation at the 5′ end of the differentiation-linked TF-encoding gene *TBX20.*** These DMRs are observed even though the gene is fully repressed in normal aorta and most postnatal tissues. (A) RefSeq gene isoforms and chromatin state segmentation (chr7:35,240,087-35,304,318). (B) Tissue-specific and atherosclerosis-associated DMRs. (C) Bisulfite-seq DNA methylation profiles. Note that the uppermost chromatin state track is from heart, one of the few *TBX20*-expressing postnatal tissues (Table 5).

Tables 1–5 (included as Supplementary files) show statistically significant atherosclerosis-associated DMRs (atheroDMRs), their over-representation among enhancers and super-enhancers, the functional associations of their linked genes based on gene ontology (GO) analysis, a literature survey of the involvement of DMR-linked genes to atherosclerosis, and RNA-seq data for the tissue-specific expression of these genes. In addition, data are presented for specific examples of genes in which athero DMRs only partially overlap leukocyte-associated DMRs (Fig. 1, Fig. 2) or overlap aorta-related enhancer and super-enhancer chromatin in genes important for different aspects of proper smooth muscle cell function (Fig. 3, Fig. 4, Fig. 5, Fig. 6). Lastly, the very strong relationship of athero DMRs to developmental transcription factor-encoding *HOX* and *TBX* genes is illustrated (Fig. 7, Fig. 8, Fig. 9, Fig. 10, Fig. 11).

## 2 Experimental design, materials and methods

### 2.1 Bioinformatics

For the atherosclerotic and control aorta samples from the same individual (88 yo female, athero aorta A, aortic arch, and control aorta A, thoracic aorta, respectively), the whole genome bisulfite sequencing (bisulfite-seq) data from Zaina et al. [2] were used. In addition, we used bisulfite-seq profiles from two additional control aorta samples: control aortas B, 34 yo male, and control aorta C, a 30 yo female; Roadmap Epigenetics Project [3], [4]. The subsection of aorta for control aorta C is not known but control aorta B, which was used for bisulfite-seq profiles and the analyzed Roadmap histone modification and chromatin segmentation profiles, was intra-abdominal aorta from below the renal arteries, before the iliac bifurcation. The Roadmap databases including the bisulfite-seq methylomes and chromatin state segmentation (chromHMM, AuxilliaryHMM) profiles are available at hubs for the UCSC Genome Browser hg19 [5] and are as previously described [6]. Chromatin state segmentation is based upon histone modifications (histone H3 lysine-4 mono-or-trimethylation; H3 lysine-27 acetylation or trimethylation; H3 lysine-36 trimethylation and H3 lysine-9 trimethylation). For the DNA methylation analysis, we found similar coverage in the DNA-seq analysis of the bisulfite-treated matched atherosclerotic and control samples. Note that the Roadmap sample labeled “macrophage” in the bisulfite-seq track at the UCSC Genome Browser [5] is actually primary CD14^+^ monocytes from blood, like the corresponding chromatin segmentation track [3]. The skeletal muscle (SkM) sample for bisulfite-seq and chromatin state was psoas muscle. The color code for the 18-state chromatin state segmentation was slightly simplified from the original [3]. Quantification of RNA-seq for tissues was from the GTEx database using transcripts per million read (TPM for Table 5) values from more than 100 samples for each tissue type [7]; the aorta tissue used for these RNA-seq analyses was from the thoracic region. Functional associations of DMRs used the Genomic Regions Enrichment of Annotations Tool, GREAT [8] and associations of the genes themselves used Database for Annotation, Visualization and Integrated Discovery, DAVID [9]. Aorta super-enhancers were determined from the dbSuper database [10].

### 2.2 Determination of athero and tissue-specific DMRs

Bisulfite-seq data from the athero aorta A and control aorta A samples were initially merged and analyzed on a site-by-site basis by applying Fisher's exact test to the counts of methylated and unmethylated CpG reads in each sample to produce site-specific p-values *p*_*i*_. Based on these results, candidate DMRs were then identified by determining the joint probability of a sequence of five or more consecutive *p*-values (*p*_*i,*_ *p*_*i+1, …,*_*p*_*i+k*_) according to the Uniform Product (UP) distribution as described in our previous study [11], where each candidate DMR was required to begin and end with a statistically significant site. This analysis identified statistically significant regions at the 0.05 level, and these were subsequently filtered to include only those regions with an average percent methylation difference (PMD) of at least ±20%, length greater than 250bp, and no gaps >200 bp between consecutive sites. Next, these samples were merged with control aorta samples B and C, using custom scripts to correct for single-bp shifts due to variable pre-processing routines. For all sites present in all four samples, logistic regression models were fit to the counts of methylated and unmethylated reads at each CpG site to determine the statistical significance associated with the difference in percent methylation between the athero aorta A and the three aorta controls. Associated *p*-values for the comparison of athero aorta vs. three controls were then analyzed using our UP method to identify candidate DMRs, which were subsequently filtered as above for length, PMD, and gaps. Our final set of athero-associated DMRs were determined from those DMRs identified both in athero A vs. control aorta A as well as in the more general comparison of athero A vs. control aortas A, B, and C. PMD values are reported based on the differences observed for athero A vs. control aorta A.

To determine tissue-specific effects among the selected Roadmap samples (aorta, left ventricle, SkM, lung, adipose, and monocyte), “one-to-many” comparisons were run in which logistic regression models were fit to determine the PM differences associated with a selected sample relative to the others as a group. Because not all sites were present for all six samples, we required that any tested site be contained in the target sample and at least four of the five non-target samples. DMR identification and filtering was done using the same approach as for the athero DMR analyses. All preprocessing and analysis was performed in R version 3.4.4 [12]using custom scripts. For identifying overlaps of athero DMRs with a normal tissue DMRs, we used an R script for each of four normal tissues (aorta, monocytes, SkM and heart) and then for each athero DMR to determine any overlap of the athero DMRs of ≥50 bp with a given tissue DMR.

### 2.3 Mapping DMRs to genes and enhancer chromatin

Scripts in R language [12] with adaptations [13], [14] were used to determine which genes and enhancer chromatin (enh chromatin) segments were associated with athero DMRs. To determine the gene isoform associated with a given DMR, the reFlat table was used [5] for the RefSeq hg19 genome (accessed January 7, 2018). The protein-coding gene or, secondarily, the non-protein-coding gene was selected with the largest overlap of the gene regions in the following order of precedence with distances given relative to the transcription start site (TSS) or the mRNA-determined transcription end sequence (TES): TSS – 5 kb to TSS + 5 kb; TSS + 5 kb to the TES; intergenic (other sequences). For determining chromatin enhancer segments overlaps, DMRs were used that had at least a 50-bp overlap total with enh chromatin from any of the following enhancer states in the 18-state chromatin segmentation model [3], [5]: state numbers 3, 8, 9 or 10.
