# Supplementary material for: Data showing atherosclerosis-associated differentially methylated regions are often at enhancers
Source: Data Brief. 2019 Mar 7;23:103812. doi: 10.1016/j.dib.2019.103812 (PMC6660590; doi:10.1016/j.dib.2019.103812)
Supplement: Multimedia component 1 [file mmc1.docx]

Competing interests: The authors declare that they have no competing interests.
